# PINK1 deficiency impairs adult neurogenesis of dopaminergic neurons

**DOI:** 10.1038/s41598-021-84278-7

**Published:** 2021-03-23

**Authors:** Sarah J. Brown, Ibrahim Boussaad, Javier Jarazo, Julia C. Fitzgerald, Paul Antony, Marcus Keatinge, Janna Blechman, Jens C. Schwamborn, Rejko Krüger, Marysia Placzek, Oliver Bandmann

**Affiliations:** 1 Bateson Centre, University of Sheffield, Sheffield, UK; 2 Department of Biomedical Science, University of Sheffield, Sheffield, UK; 3 Department of Neuroscience, Sheffield Institute for Translational Neuroscience (SITraN), The University of Sheffield, 385a Glossop Road, Sheffield, S10 2HQ UK; 4 Centre for Discovery Brain Science, University of Edinburgh, Edinburgh, Scotland; 5 Translational Neuroscience, Luxembourg Centre for Systems Biomedicine, University of Luxembourg, Luxembourg, Luxembourg; 6 Disease Modelling and Screening Platform (DMSP), Luxembourg Centre of Systems Biomedicine, University of Luxembourg & Luxembourg Institute of Health, Luxembourg, Luxembourg; 7 Developmental Biology, Luxembourg Centre for Systems Biomedicine, University of Luxembourg, Luxembourg, Luxembourg; 8 OrganoTherapeutics SARL, Luxembourg, Luxembourg; 9 Hertie-Institute for Clinical Brain Research, University of Tübingen, Tübingen, Germany; 10 Weizmann Institute of Science, Rehovot, Israel; 11 Parkinson Research Clinic, Centre Hospitalier de Luxembourg (CHL), Luxembourg, Luxembourg; 12 Transversal Translational Medicine, Luxembourg Institute of Health (LIH), Luxembourg, Luxembourg

**Keywords:** Parkinson's disease, Parkinson's disease

## Abstract

Recent evidence suggests neurogenesis is on-going throughout life but the relevance of these findings for neurodegenerative disorders such as Parkinson’s disease (PD) is poorly understood. Biallelic *PINK1* mutations cause early onset, Mendelian inherited PD. We studied the effect of PINK1 deficiency on adult neurogenesis of dopaminergic (DA) neurons in two complementary model systems. Zebrafish are a widely-used model to study neurogenesis in development and through adulthood. Using EdU analyses and lineage-tracing studies, we first demonstrate that a subset of ascending DA neurons and adjacent local-projecting DA neurons are each generated into adulthood in wild type zebrafish at a rate that decreases with age. Pink1-deficiency impedes DA neurogenesis in these populations, most significantly in early adult life. Pink1 already exerts an early effect on Th1^+^ progenitor cells rather than on differentiated DA neurons only. In addition, we investigate the effect of PINK1 deficiency in a human isogenic organoid model. Global neuronal differentiation in PINK1-deficient organoids and isogenic controls is similar, but PINK1-deficient organoids display impeded DA neurogenesis. The observation of impaired adult dopaminergic neurogenesis in Pink1 deficiency in two complementing model systems may have significant consequences for future therapeutic approaches in human PD patients with biallelic *PINK1* mutations.

## Introduction

Parkinson’s disease (PD) is a relentlessly progressive neurodegenerative disorder. Its pathological hallmark is the loss of dopaminergic (DA) neurons in the substantia nigra pars compacta (SNpc) of the midbrain. The causes and mechanisms underlying the observed cell loss remain poorly understood.

Autosomal recessively inherited mutations in the *PINK1* gene typically cause early onset PD^1,2^. PINK1 has been implicated in the regulation of mitophagy, mitochondrial function and oxidative stress^3–9^, but as yet, the underlying mechanisms of PINK1-mediated PD are not fully understood. *Pink1* knockout mice do not display reductions in DA neurons in the substantia nigra^10^. In contrast, PINK1 deficiency in zebrafish results in both reduced numbers of DA neurons in larval and adult zebrafish as well as impaired mitochondrial function and morphology^8^.

The finding that neurons can be generated in the adult brain^11^, together with the observation that Parkinson’s disease genes, including *SNCA, PINK1* and *LRRK2*, are implicated in the regulation of neurogenesis^12–19^, raises the possibility that reduced de novo neurogenesis from neural stem/progenitor cells in adult life could contribute to neuronal decline in Parkinson’s disease. As yet, no study has definitively addressed whether DA neurons are added to ascending dopaminergic populations throughout adult life, but there is evidence that mammalian adult DA neuronal generation can be triggered through stimulation^20–22^. A key unanswered question is whether ascending adult DA neurons or other neuronal populations that could play a role in disease pathogenesis can be generated and/or turned-over in adulthood. This is an important question, as mechanisms involved in de novo adult neurogenesis could be susceptible to ageing-related processes or influenced by the deleterious effect of PD genes and thus account for, or at least contribute to, a subset of PD subtypes.

Zebrafish are a particularly valuable tool to study neurogenesis in vertebrates. Basal levels of neurogenesis occur at higher levels than in mammals, and additional proliferative zones are found throughout the brain^23,24^. One such proliferative zone, termed the rostral posterior tuberculum (PT), harbours distinct populations of DA neurons. Retrograde dye tracing studies show that three of these (a population of small neurons of the periventricular nucleus of the posterior tuberculum (TPp) and two populations of magnocellular neurons) ascend to the subpallium and are therefore thought to correspond to mesostriatal systems in the mammalian brain. Immediately ventral to these are locally-projecting DA neurons of the paraventricular organ (PVO)^25–28^. Previous studies have suggested that embryonic generation of ascending DA neurons in the PT is complete by 30 h post-fertilisation (hpf)^29^ and that embryonic populations termed DC1 and DC3 may give rise to adult TPp and PVO DA neurons, respectively^26^.

Here we first examine the character and generation of DA neurons in the zebrafish PT throughout life to address whether any population is generated in adulthood. We build on previous reports that describe the three ascending DA populations in the PT, to show that these express the transcription factor Otp, which distinguishes them from adjacent local-projecting DA neurons. Using EdU analyses and lineage-tracing studies, we demonstrate that ascending TPp DA neurons and local-projecting PVO neurons, but not magnocellular ascending DA neurons, are each generated into adulthood in wild type animals at a rate that decreases with age. Crucially, PINK1-deficiency diminishes DA neurogenesis in early adult life of *pink1*^-/-^ zebrafish^8^. We subsequently studied the role of PINK1 on DA neurogenesis in a human organoid model and report a dramatic, selective effect of PINK1 deficiency on DA neurons in a *PINK1* deficient human isogenic organoid model.

## Results

### Dopaminergic populations in the zebrafish posterior tuberculum

**Figure 1:**
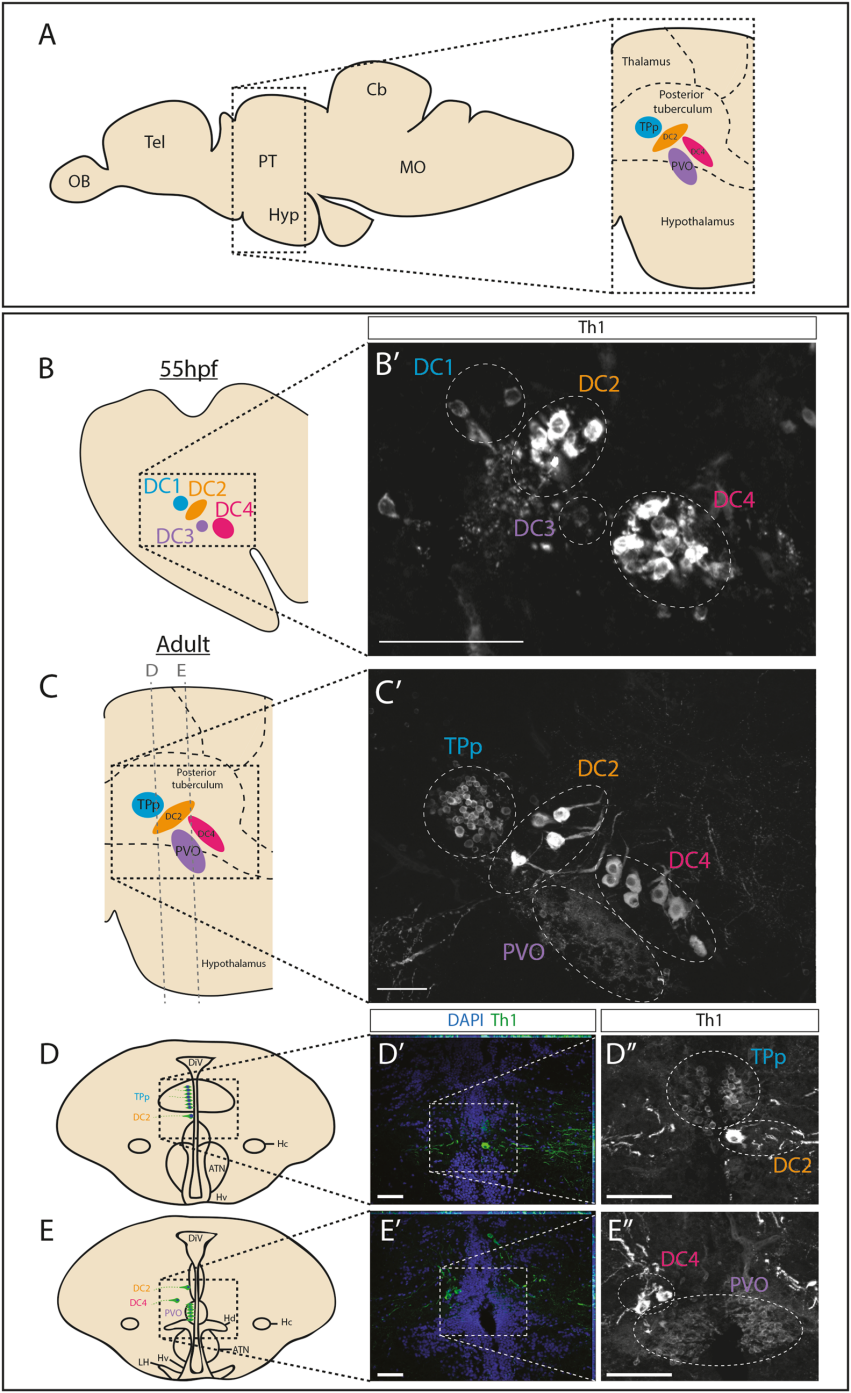
DA populations in the embryonic and adult zebrafish posterior tuberculum (PT). (**A**) Schematic side view of an adult zebrafish brain. The boxed region indicates location of the PT and highlights the DA populations examined in this study (right) (TPp in blue, DC2 in orange, PVO in purple, DC4 in magenta). (**B–C**′) DA neurons can be assigned to particular populations on the basis of size and position: DC1/TPp, DC2, DC3/PVO and DC4. (**B**) Schematic showing DC1 (blue), DC2 (orange), DC3 (purple) and DC4 (magenta) DA populations of the embryonic PT. (**B**′) Immunohistochemical analysis for Th1 in a representative sagittal section taken through a wild type 55hpf zebrafish brain. DA populations are circled with a dotted line and labelled with colour coding outlined in (**B**). (**C**) Schematic showing the TPp (blue), DC2 (orange), PVO (purple) and DC4 (magenta) DA populations of the adult PT. Lines indicate planes of sections shown in (**D**–**E**″). (**C**′) Immunohistochemical analysis for Th1 in a representative sagittal section taken through a wild type 12 month brain. DA populations are circled with a dotted line and labelled with colour coding outlined in (**A**,**C**). DC2-derived neurites can be detected ventral to DAPI-stained DC2 nuclei. (**D–E**″) DA populations in the adult zebrafish brain in transverse sections allow robust quantification of adult populations. Schematics of transverse sections taken through an adult zebrafish brain showing DA neurons (green) in the TPp and DC2 populations (**D**) or in PVO and DC4 populations (**E**). Immunohistochemical analyses for Th1 (green) with DAPI counter-staining (blue) shows the TPp and DC2 populations (**D**′) and the PVO, DC2 and DC4 populations (**E**′). (**D**″,**E**″) Magnified image of boxed regions in schematics (**D**′,**E**′) shown without DAPI. DA populations are circled with a dotted line and labeled with colour coding outlined in (**A**,**C**). Yellow dashed line indicates ventricle. Scale bars: 50 μm. Schematics in (**D**,**E**) are based on anatomical drawings by Rink and Wullimann^25^.

The rostral PT lies dorsal to, and overlaps with the hypothalamus^25,26,28^; Fig. 1A]. Previous reports have characterised its resident DA neuronal populations in adulthood and have suggested that these arise from defined embryonic populations. We first confirmed and extended these reports. Immunohistochemical analysis for tyrosine hydroxylase-1 (Th1) in sagittal sections through 55hpf zebrafish embryos confirms the distinct DA populations that can be identified through position, size and intensity of Th1-labelling. DC1 and DC3 populations are composed of small (parvocellular), weakly-labelling neurons; large (magnocellular) DC2 neurons lie between DC1 and DC3 populations, and a second magnocellular population, DC4, overlaps with and lies posterior to DC3 (Fig. 1B, B′; Supplementary Fig. 1A; Supplementary Video 1). In the adult, Th1^+^ neuronal populations occupy similar relative positions and show similar relative size to those in the embryo^25,26,^. Analysis of sagittal sections through 12 month zebrafish reveals the parvocellular DA neurons of the TPp, the parvocellular DA neurons of the PVO and two populations of magnocellular neurons that bear similarity, in position and morphology, to DC2 and DC4 embryonic populations; we term these DC2^A^ and DC4^A^ (where ^A^ refers to the adult population) (Fig. 1C, C′; Supplementary Fig. 1A; Supplementary Video 2).

To enable better resolution of individual neurons, we analysed Th1^+^ immunoreactivity in transverse sections. The morphology of the diencephalic ventricle of the PT shows characteristic features along the rostro-caudal and dorso-ventral axes^25,28^. This, and the distinctive morphology of each, enables accurate assignment of Th1^+^ neurons: small round DC1 and TPp neurons that lie at and beyond the ventricle; large DC2 and DC2^A^ neurons that lie at the edge of the ventricular zone; bipolar DC3 and PVO neurons at the ventricle; large DC4 and DC4^A^ populations that lie well-beyond the ventricle. (Fig. 1D,E″; Supplementary Fig. 1; Supplementary Fig. 2; Supplementary Videos 3–7).

**Figure 2:**
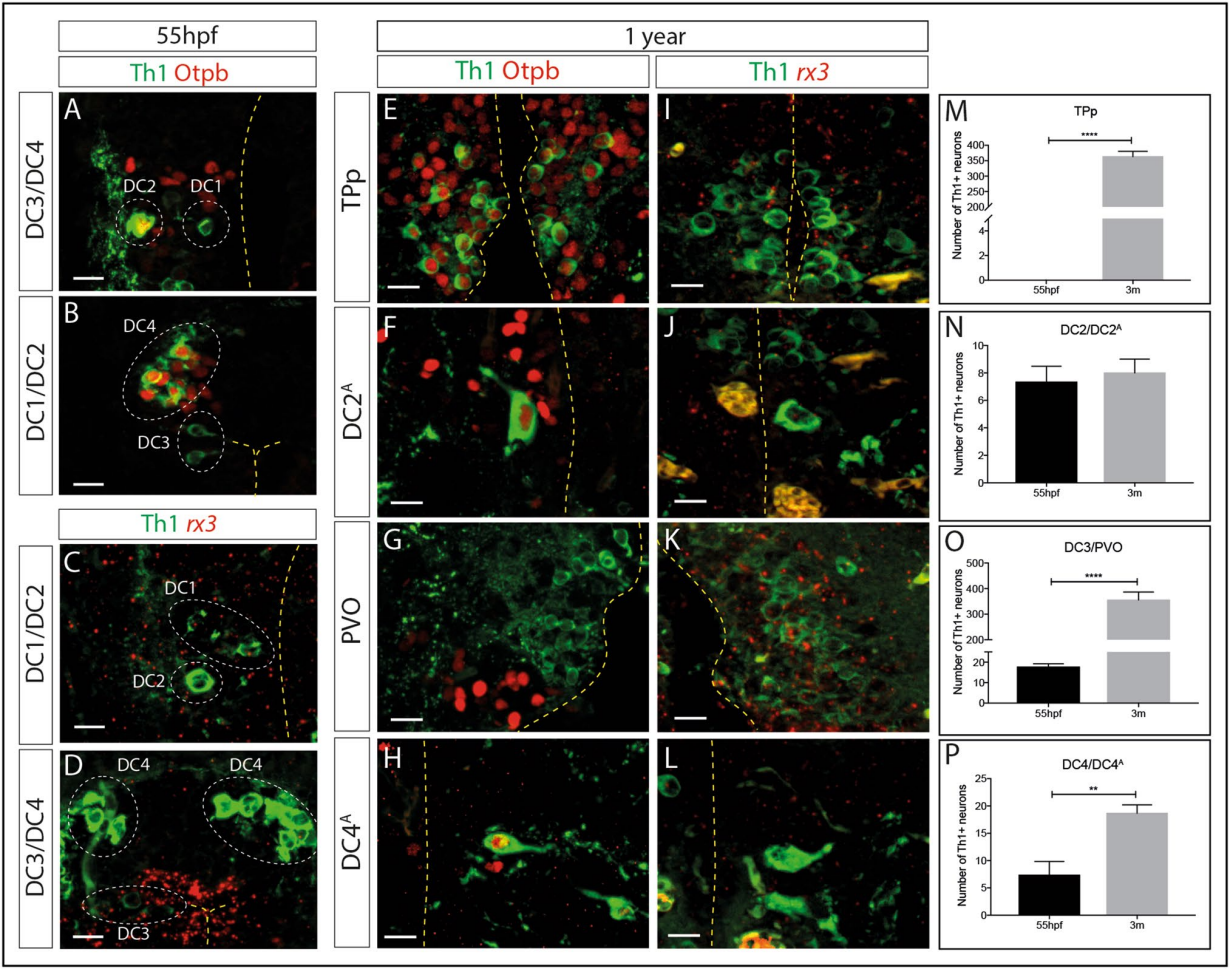
Expression of Otp and *rx3* in the embryonic and adult zebrafish posterior tuberculum (PT). (**A-L**) Immunohistochemical analysis for Th1 (green) and Otp (red) in representative transverse sections of 55hpf (**A**,**B**) or 12 month old (**E**–**H**) wild type zebrafish, or immunohistochemical analysis for Th1 (green) and *rx3* (red) in transverse sections of 55hpf (**C**,**D**) or 12-month old (**I**–**L**) wild type zebrafish. Otp^+^Th1^+^ DA neurons are detected in DC2 (**B**) and DC4 (**D**) populations in the 55hpf embryo, whereas DA neurons in DC1 (**A**) and DC3 (**C**) populations are not Otp^+^. In the 12 month old zebrafish, Otp^+^Th1^+^ DA neurons are detected in the TPp (**E**), DC2^A^ (**F**) and DC4^A^ (**H**) populations but DA neurons in the PVO are not Otp^+^ (**G**). *rx3*^+^ is highly expressed in DC3 (**D**) neurons but barely detected in DC1, DC2 (**C**) or DC4 (**D**) neurons in the 55hpf embryo. In 12 month old zebrafish, *rx3* is detected in PVO DA neurons (**K**) but barely detected in TPp (**I**), DC2^A^ (**J**) or DC4^A^ (**L**) DA neurons. Yellow dashed line marks the ventricle. Scale bars: 10 μm. (**M–P**) Quantitative analyses across entire populations show that the number of Th1^+^ DA neurons significantly increases between 55hpf and 3 months of age in the TPp (**M**) (*t*-test; *p* =  < 0.0001, n = 3 fish each) (as described in the text, we propose TPp DA neurons are not equivalent to DC1 DA neurons, therefore at 55hpf we started the TPp DA quantification at zero), the PVO (**N**) (*t*-test; *p* =  < 0.0001, n = 3 fish each) and the DC4 population (**P**) (*t*-test; *p* = 0.0026, n = 3 fish each). The number of DA neurons in the DC2 population does not significantly increase between 55hpf and 3 months (**N**) (*t*-test; *p* = 0.4918, n = 3 fish each).

Previous reports have indicated that the transcription factors Otp and Rx3 are required for the specification and/or differentiation of embryonic ventral diencephalic DA neurons^30,31^ and we therefore examined expression of each, relative to DA neurons (Fig. 2A–D). In the embryo, Otp is selectively expressed in DC2 and DC4 neurons at 55 hpf, but not in DC1 or DC3 neurons (Fig. 2A,B) and *rx3* is expressed at high levels in DC3 neurons, but barely detected in DC1, DC2 or DC4 populations (Fig. 2C,D). In the 12 month adult brain, Otp is expressed in DC2^A^ and DC4^A^ neurons (Fig. 2F,H), strengthening the idea that these are related to the DC2 and DC4 embryonic populations. However, in contrast to embryonic DC1 DA neurons, adult TPp DA neurons all co-express Otp (Fig. 2E). This suggests that the embryonic DC1 population is not the same as the adult TPp population. *Rx3,* but not Otp*,* is expressed at high levels in PVO neurons (Fig. 2G,K), adding weight to the suggestion that DC3 embryonic neurons are related to adult PVO neurons.

In summary, these analyses suggest two conclusions. First, similarities in morphology, position and Otp expression suggests that DC2/DC2^A^ populations, DC3/PVO populations and DC4/DC4^A^ populations are related. In contrast, the difference in expression of Otp suggests that DC1/TPp populations may not be related, and instead that the adult Otp^+^ TPp population is generated later than 55hpf. Second, in adulthood, expression of Otp is confined to ascending neurons in the dorsal PT (TPp, DC2^A^ and DC4^A^) whereas strong *rx3* expression is confined to local-projecting PVO neurons of the ventral PT/dorsal hypothalamus.

### Dopaminergic neurons of the posterior tuberculum increase in number over time

We next determined whether the number of DA neurons in the PT increases between 55hpf and 3 months (young adult) and if so, whether different DA neuronal subpopulations expand at distinct time points. This revealed that the number of DA neurons markedly increased over time in three of the four subsets of DA neurons: (TPp (Fig. 2M); DC3/PVO (Fig. 2O); DC4/DC4^A^ (Fig. 2P), but not in the DC2/DC2^A^ population (Fig. 2N). This quantification also revealed marked differences in population size between adult PT DA neuronal populations: the TPp and PVO populations contain several hundred DA neurons but the DC2^A^ and DC4^A^ populations are much smaller with only ~ 10–25 DA neurons (Fig. 2M–P).

### Dopaminergic neurons in the TPp and the PVO are generated in adult life, but generation declines with age

**Figure 3:**
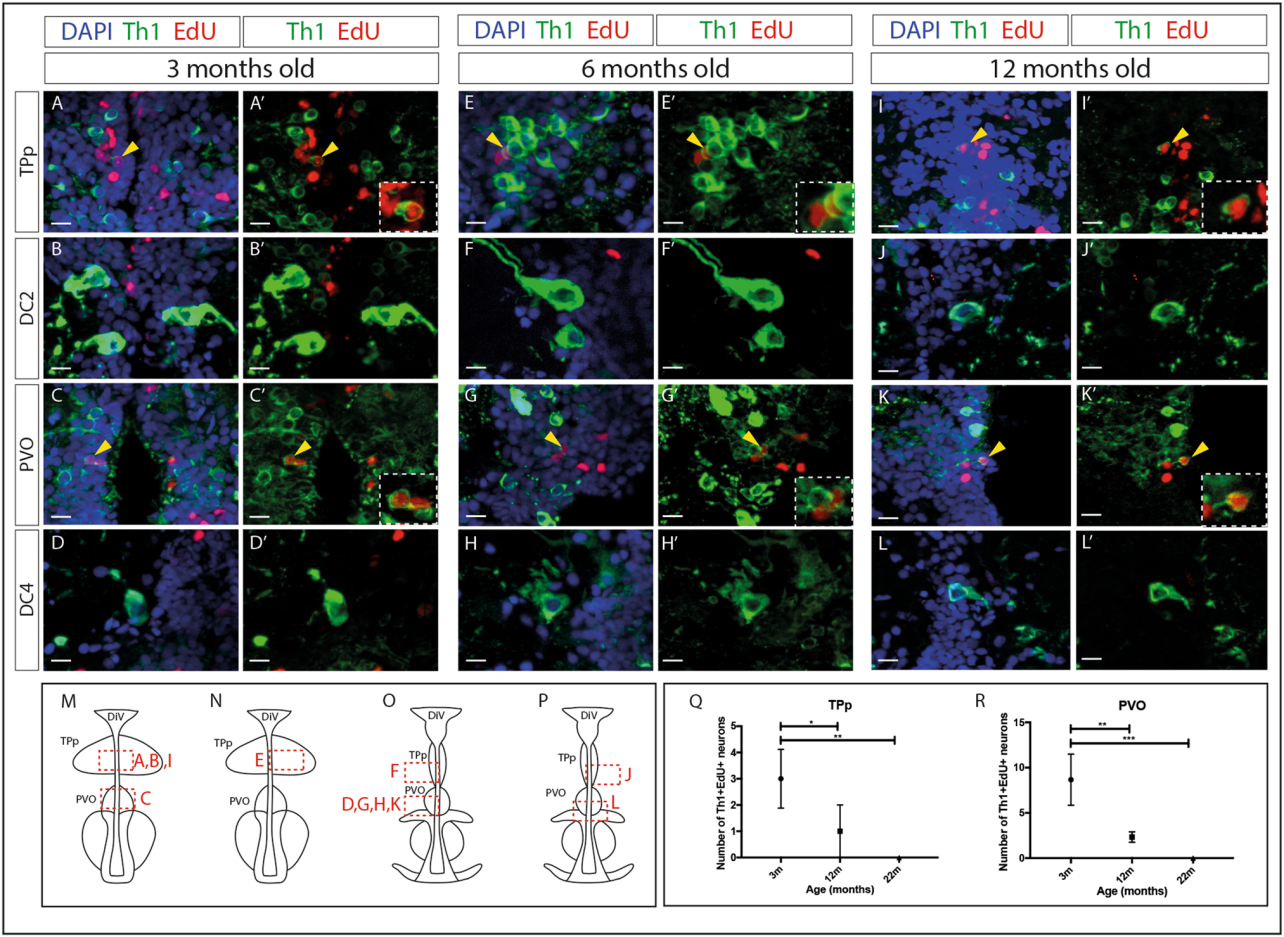
DA neurons are generated in the PT in adulthood, but generation decreases with age. (**A–L**′) Immunohistochemical analysis for Th1 (green), ClickIT™ labeling for EdU (red) and counterstained with DAPI (blue) (**A**,**B**,**C**,**D**,**E**,**F**,**G**,**H**,**I**,**J**,**K**,**L**) or shown without DAPI (**A**′,**B**′,**C**′,**D**′,**E**′,**F**′,**G**′,**H**′,**I**′,**J**′,**K**′,**L**′) in representative transverse sections of 3 month (**A**–**D**′)(n = 9 fish), 6 month (**E**–**H**′)(n = 2 fish) or 12 month (**I**–**L**′)(n = 3 fish) wild type zebrafish brains. Th1^+^EdU^+^ cells are detected in the TPp in 3 month (**A**,**A**′), 6 month (**E**,**E**′) and 12 month (**I**,**I**′) old brains, and in the PVO of 3 month (**C**,**C**′), 6 month (**G**,**G**′) and 12 month (**K**,**K**′) old brains. No EdU labelling was detected in DC2 neurons (**B**,**B**′,**F**,**F**′,**J**,**J**′) or DC4 neurons (**D**,**D**′,**H**,**H**′,**L**,**L**′). Yellow arrowheads point to double-positive cells and insets show magnified image of double-labeled cells. Yellow dashed line indicates ventricle. Scale bars: 10 μm. (**M–P**) Schematics indicating the position of images (**A**–**L**′) within the PT, red boxes indicate position of the corresponding image in relation to the ventricle. Images in (**A**–**L**′) are representative of sections across the entire A–P extent of each population; those chosen best highlight the distinct morphology of each neuronal population. (**Q**) Quantitative analyses through the entire population shows that the number of Th1^+^EdU^+^ cells in the TPp is decreased in 12 month fish (n = 3 fish) compared to 3 month fish (n = 9 fish) (two-way ANOVA, *p* = 0.0277), and further decreased in 22 month fish (n = 3 fish) compared to 3 month fish (two-way ANOVA, p = 0.0019). (**R**) The number of Th1^+^EdU^+^ cells in the PVO is decreased in 12 month fish (n = 3 fish) compared to 3 month fish (n = 9 fish) (two-way ANOVA, *p* = 0.0039), and further decreased in 22 month fish (n = 3 fish) compared to 3 month fish (two-way ANOVA, *p* = 0.0003). Schematic in (**M**–**P**) is based on anatomical drawings by Rink and Wullimann^25^.

DA neurons in the zebrafish caudal hypothalamus can be generated in adulthood, but as yet no study has examined whether adult DA neurogenesis also occurs in the rostral PT. To address this we performed EdU pulse-chase analyses in 3 month old fish. We identified Th1^+^EdU^+^ neurons in both the TPp (Fig. 3A,A′,M; Supplementary Video 8) and the PVO (Fig. 3C,C′,M; Supplementary Video 9) (approximately 1% and 2.5% of total DA neuron numbers in the TPp and the PVO respectively) but not in the DC2^A^ or DC4^A^ populations (Fig. 3B,B′,D,D′,M,O). In conclusion, these studies show de novo generation of two populations of parvocellular neurons in the adult rostral PT, ascending Otp^+^Th1^+^ DA neurons of the TPp, and local-projecting *rx3*^+^Th1^+^ DA neurons of the PVO, but not of magnocellular ascending DA neuronal populations.

To gain a representative view of de novo neurogenesis across the lifespan, we performed similar pulse-chase analyses at 6-, 12- and 22 months of age. At both 6- and 12 months, EdU^+^Th1^+^ neurons were detected in the TPp (Figs. 3E,E′,N; I,I′,M) and the PVO (Fig. 3G,G′,O; K,K′,O) but not in the DC2^A^ (Fig. 3F,F′,O; J,J′,P) or DC4^A^ (Fig. 3H,H′,O; L,L′,P) populations. At 22 months of age, no Th1^+^EdU^+^ neurons could be detected in any population (Supplementary Fig. 3), suggesting an age-related decline in DA neurogenesis. To confirm this, we quantified EdU^+^ Th1^+^ neurons in the TPp (Fig. 3Q) and the PVO (Fig. 3R) at 3-, 12- and 22 months of age. This showed that in both populations, the number of newly-generated DA neurons significantly decreased between 3- and 12 months of age and between 3- and 22 months of age (Fig. 3Q,R).

**Figure 4:**
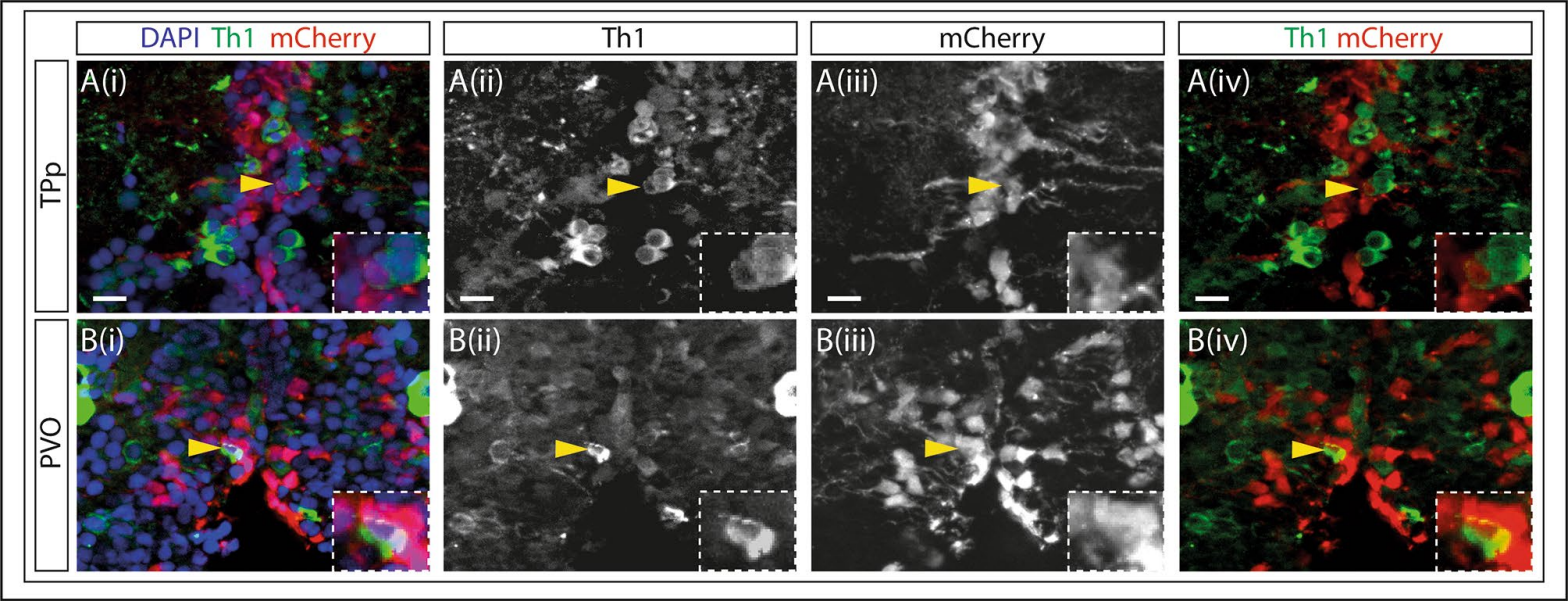
DA neurons in the adult PVO are generated from Her4-expressing progenitors. (**A–B**) Fluorescent MIP of the PT of double transgenic (Tg(her4:ERT2CreERT2); Tg(ubi:loxGFPloxmCherry)) 3 month zebrafish injected with tamoxifen. Cell nuclei are labelled with DAPI (blue), DA neurons with Th1 (green) and Her4 progenitors with mCherry (red) throughout. Th1 is shown as a single channel in (ii), mCherry is shown in (iii). Th1 (green) and mCherry (red) are shown together in (iv). Boxed regions show magnified image of the double labelled cell. (**Ai–iv**) TPp: mCherry and Th1 mark adjacent cells but are not co-localised (yellow arrows). (**Bi–iv**) PVO: Double staining shows co-localisation of mCherry and Th1. The presence of mCherry + Th1 + cells in the PVO suggests that newly generated DA neurons are derived from Her4 progenitors in this neuronal subpopulation. Yellow dashed line indicates ventricle.

To provide independent confirmation that DA neurons can be newly-generated in the adult PT from neural stem/progenitor cells, we conditionally lineage-traced *her4-*expressing progenitors in the 3-month PT^32–36^: *her4* is expressed in progenitor-like radial glial cells cells immediately adjacent to the PT^37^ and we therefore reasoned that DA neurons may be generated from them. In recombination-induced Tg(her4:ERT2CreERT2); Tg(ubi:loxGFPloxmCherry) fish, mCherry^+^Th1^+^ DA neurons were detected in the PVO but not in the TPp (Fig. 4A,B; Supplementary Videos 10–13). mCherry^+^Th1^+^ DA neurons were also detected in the caudal hypothalamus (Supplementary Fig. 4; Supplementary Videos 14 and 15).

Together, our data shows for the first time, that DA neurons are added to the ascending TPp population and the locally-projecting PVO population and that the rate of DA generation in the TPp and the PVO decreases with age.

### PINK1 deficiency reduces adult neurogenesis in *pink*^-/-^ zebrafish

Previous studies have linked PINK1 to the regulation of adult neurogenesis^17,18,38,39^ but no study has yet investigated the role of PINK1 specifically in DA neuronal generation. Using ENU mutagenesis, we previously made a *pink1*^-/-^ zebrafish line with a stop mutation in the PINK1 kinase domain resulting in abolished kinase activity([8]).

**Figure 5:**
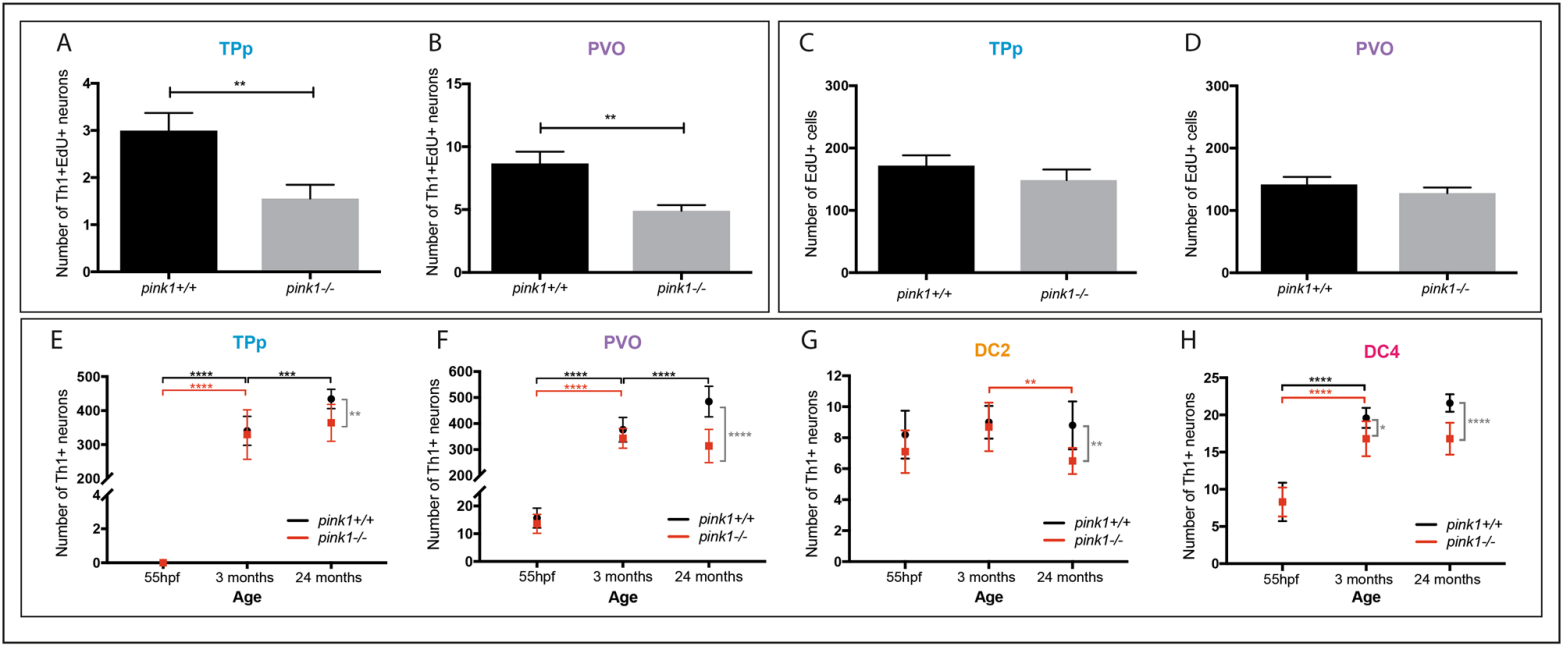
Adult generation of DA neurons is impeded in *pink1*^-/-^ zebrafish. (A,B) Quantification of the number of newly generated Th1^+^EdU^+^ DA neurons in *pink1*^+/+^ and *pink1*^-/-^ zebrafish shows a significant reduction in the TPp (A) (*t*-test, *p* = 0.0078, n = 9 for each) and in the PVO (B) (*t-*test, *p* = 0.0017, n = 9 fish for each) of 3 month *pink1*-/- zebrafish compared to *pink1* + / + siblings. (**C,D**) No difference in the total number of EdU^+^ cells in the TPp (C) (*t*-test, *p* = 0.3431, n = 9 fish for each) and in the PVO (D) (*t*-test, *p* = 0.3640, n = 9 fish for each) in 3 month *pink1*-/- zebrafish compared to *pink1*^+/+^ siblings. (**E**) Quantification of DA neurons in the TPp shows an increase in the number of DA neurons in *pink1*^+/+^ zebrafish (black line) between 55hpf and 3 months (two-way ANOVA, *p* =  < 0.001, n = 10 fish for each) and between 3- and 24 months (two-way ANOVA, *p* = 0.0001, n = 10 fish for each). In *pink1*^-/-^ zebrafish, the number of DA neurons increases between 55hpf and 3 months (two-way ANOVA, *p* =  < 0.001, n = 10 fish for each), but not between 3- and 24 months (two-way ANOVA, *p* = 0.4587, n = 10 fish for each). *pink1*^-/-^ zebrafish (red line) have significantly fewer DA neurons in the TPp at 24 months (two-way ANOVA, *p* = 0.0065, n = 10 fish for each) but not at 55hpf (two-way ANOVA, *p* =  > 0.9999, n = 10 fish for each) or at 3 months (two-way ANOVA, *p* = 0.9917, n = 10 fish for each). (**F**) Quantification of DA neurons in the PVO shows in *pink1*^+/+^ zebrafish (black line), the number of DA neurons significantly increases between 55hpf and 3 months (two-way ANOVA, *p* =  < 0.0001, n = 10 fish for each) and between 3 and 24 months (two-way ANOVA, *p* =  < 0.0001, n = 10 fish for each). In *pink1*^-/-^ zebrafish (red line), the number of DA neurons significantly increases between 55hpf and 3 months (two-way ANOVA, *p* =  < 0.0001, n = 10 fish for each) but not between 3 and 24 months. *pink1*^-/-^ zebrafish have significantly fewer DA neurons than *pink1*^+/+^ zebrafish in the PVO at 24 months of age (two-way ANOVA, *p* =  < 0.0001, n = 10 fish for each) fish fish fish fish fish fish fish fish fish fish fish eachfish eachfish eachfish each(**G**) Quantification of DA neurons in the DC2 population shows *pink1*^+/+^ zebrafish (black line), the number of DA neurons does not significantly increase between 55hpf and 3 months, or between 3 and 24 months. In *pink1*^-/-^ zebrafish (red line), the number of DA neurons does not significantly increase between 55hpf and 3 months, but significantly decreases between 3 and 24 months (two-way ANOVA, *p* = 0.0077, n = 10 fish for each). *pink1*^-/-^ zebrafish have significantly fewer DA neurons than *pink1* + / + zebrafish in the TPp at 2-years of age (two-way ANOVA, *p* = 0.0065, n = 10 fish for each). (**H**) Quantification of DA neurons in the DC4 population shows in *pink1*^+/+^ zebrafish (black line), the number of DA neurons significantly increases between 55hpf and 3 months (two-way ANOVA, *p* =  < 0.0001, n = 10 fish for each) but not between 3 and 24 months. In *pink1*-/- zebrafish (red line), the number of DA neurons significantly increases between 55hpf and 3 months (two-way ANOVA, *p* =  < 0.0001, n = 10 fish for each) but not between 3 and 24 months. *pink1*-/- zebrafish have significantly fewer DA neurons than *pink1*^+/+^ zebrafish in the PVO at 3 months (two-way ANOVA, *p* = 0.0308, n = 10 fish for each) and even more so at 24 months of age (two-way ANOVA, *p* =  < 0.0001, n = 10 fish for each).

We determined the effect of loss of PINK1 on DA population size over the life course, quantifying the number of Th1^+^ DA neurons in *pink1*^+/+^ and *pink1*^-/-^ zebrafish in 55hpf embryos (a time when rostral PT DA populations are beginning to be established^26,29^), 3 month, and 24 month adult fish. In *pink1*^+*/*+^ zebrafish, DA neurons are added to both the TPp and PVO between 55hpf and 3 months, and the populations are further expanded between 3 and 24 months of age (Fig. 5E,F). In *pink1*^-/-^ zebrafish, DA neurons are newly generated in similar numbers to those in wild-type fish, up to 3 months. However, neither the TPp nor the PVO population expand significantly between 3 and 24 months (Fig. 5E,F) and by 24 months, *pink1*^-/-^ fish have significantly fewer DA neurons than *pink1*^+/+^ fish in both the TPp (Fig. 5E) and the PVO (Fig. 5F). Together, these analyses show that PINK1 is required for the expansion of parvocellular DA neurons in the ascending TPp population and the local-projecting PVO population.

We next asked whether loss of PINK1 affects DC2/DC2^A^ and DC4/DC4^A^ neuronal populations which do not appear to be generated in adulthood (Fig. 2). We found no significant increase in the number of Th1^+^ DC2/DC2^A^ DA neurons in *pink1*^-/-^ or *pink1*^+/+^ zebrafish between 55hpf and 3 months. However, in contrast to *pink1*^+/+^ zebrafish, there is a significant decrease in the number of DC2/DC2^A^ DA neurons between 3 and 24 months in *pink1*^-/-^ zebrafish (Fig. 5G). The number of DC4/DC4^A^ neurons markedly increased between 55hpf and 3 months in *pink1*^+/+^ zebrafish, with a mild (non-significant) increase between 3 and 24 months (Fig. 5H). The number of DC4/DC4^A^ neurons also increased in *pink1*^-/-^ zebrafish between 55 h and 3 months but to a lesser extent than in *pink1*^+/+^ zebrafish resulting in a lower number of DA neurons with a considerably greater difference at 24 months (Fig. 5H).

The loss of PINK1 therefore leads to a reduction in neuronal populations in both DA subsets that expand in adulthood and those that do not. The reduction of the DC2^A^ population in later life stages suggests that DC2^A^ DA neurons may degenerate in zebrafish lacking functional PINK1. However, the reduction in expanding populations could occur either as a consequence of degeneration or as a consequence of reduced de novo neurogenesis. We therefore directly addressed whether loss of Pink1 inhibits neurogenesis in adult-expanding populations by analysing the generation of DA neurons in 3 month *pink1*^+/+^ and *pink1*^-/-^ zebrafish through acute EdU-labelling. The number of newly-generated Th1^+^EdU^+^ neurons is significantly decreased in both the TPp (Fig. 5A) and the PVO (Fig. 5B) in *pink1*^-/-^ zebrafish compared to *pink1*^+/+^ siblings. To assess whether loss of PINK1 results in overall reduction in proliferation, the total number of EdU^+^ cells was quantified in these areas, which also contain serotonergic neurons and glial cells rather than just DA neurons. There was no significant difference in the number of EdU^+^ cells between *pink1*^-/-^ and *pink1*^+/+^ zebrafish in the TPp (Fig. 5C) or in the PVO (Fig. 5D), suggesting that PINK1 deficiency results in reduced ability to generate DA neurons, but does not affect overall levels of proliferation in the rostral PT of adult zebrafish.

Taken together, this shows that physiologically, TPp and the PVO DA neuron populations expand with age. Zebrafish lacking functional PINK1 display comparable expansion of DA neuron populations in early life stages in the TPp and PVO, but fail to expand these populations in later stages of life. De novo neurogenesis accounts, at least in part, for the normal expansion in wild-type and *pink1*^+/+^ zebrafish. Critically, PINK1 deficiency reduces adult DA neurogenesis in *pink*^*-/-*^ zebrafish. No anatomical or morphological differences were observed between wild type and *pink*^*-/-*^ at 3 months or 2 years of age, further strengthening the conclusion that the effects of PINK1 deficiency are specific to the DA population.

### Loss of PINK1 results in a reduced number of Otp^+^ progenitors

**Figure 6:**
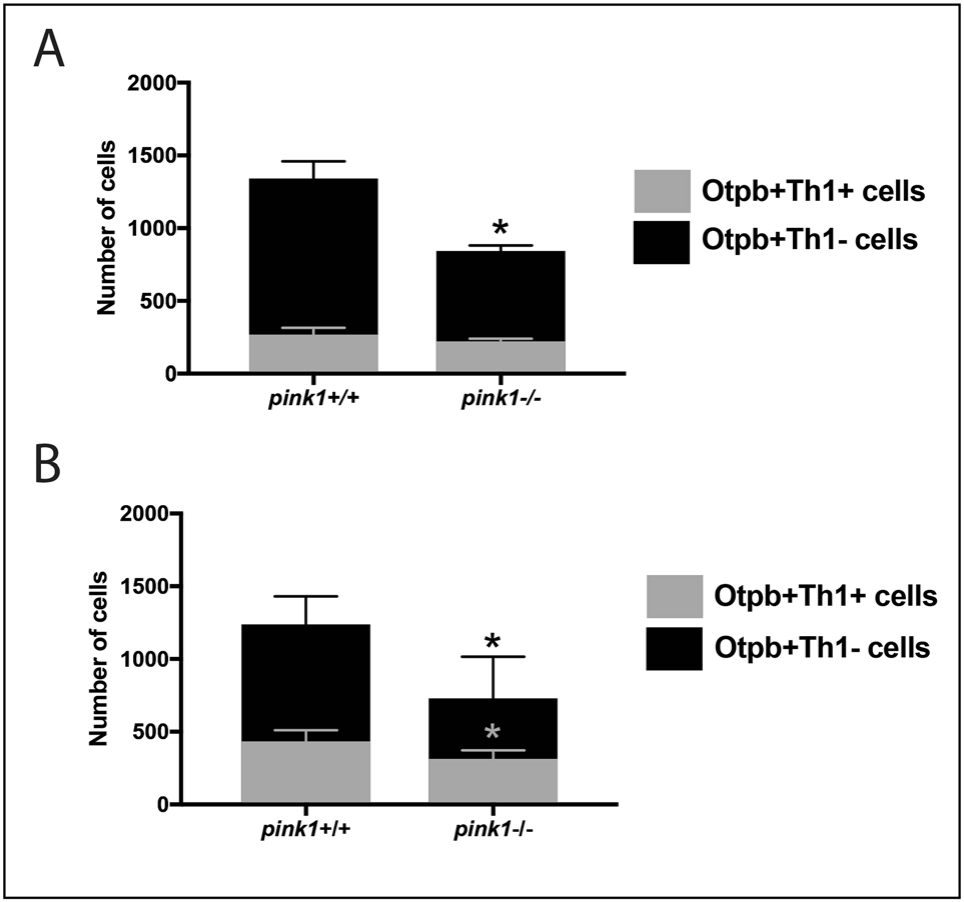
Reduced population of Otp^+^ progenitors in the TPp of *pink1*^-/-^ zebrafish at 3 months and 24 months of age. (**A**) Quantification of the number of Otp^+^Th1^-^ progenitors and the number of Otp^+^Th1^+^ DA neurons in the TPp at 3 months shows a significant reduction in the number of Otp^+^Th1^-^ progenitors in the *pink1*^-/-^ zebrafish compared to *pink1*^+/+^ zebrafish (*t*-test, *p* = 0.0292, n = 2 fish for each), but no significant difference in the number of Otp^+^Th1^+^ DA neurons**.** (**B**) Quantification of the number of Otp^+^Th1^-^ progenitors and the number of Otp^+^Th1^+^ DA neurons in the TPp at 24 months shows a significant reduction in the number of Otp^+^Th1^-^ progenitors in the *pink1*^-/-^ zebrafish compared to *pink1*^+/+^ zebrafish (*t-*test, *p* = 0.0259, n = 4 fish for each), and in the number of Otp^+^Th1^+^ DA neurons (*t-*test, *p* = 0.0453, n = 4 fish for each).

Our analyses strongly suggest that the reduction in DA neuronal number in the TPp of adult *pink1*^-/-^ zebrafish could at least partially be explained due to a decreased rate of de novo neurogenesis. We therefore finally reasoned that PINK1 may already exert an early effect on Th1^+^ progenitor cells rather than on differentiated DA neurons only. Given the observation that all Th1^+^ TPp DA neurons co-express the progenitor marker, Otp, we hypothesized that the numbers of Otp^+^Th1^-^ progenitor cells as well as the numbers of Otp^+^Th1^+^ DA neurons, will be reduced by loss of Pink1. To address this, we quantified the number of Otp^+^Th1^+^ and Otp^+^Th1^-^ neurons in the TPp in both 3 month and 2-year *pink1*^+/+^ and *pink1*^-/-^ zebrafish. This showed that at both time points, *pink1*^-/-^ zebrafish have significantly fewer Otp^+^Th1^-^ progenitor cells in the TPp (Fig. 6A,B). Therefore, loss of Pink1 results in a reduced population of Otp-expressing progenitors in both early and late adult life stages.

### PINK1 deficiency impairs dopaminergic differentiation in human midbrain-specific organoids

**Figure 7:**
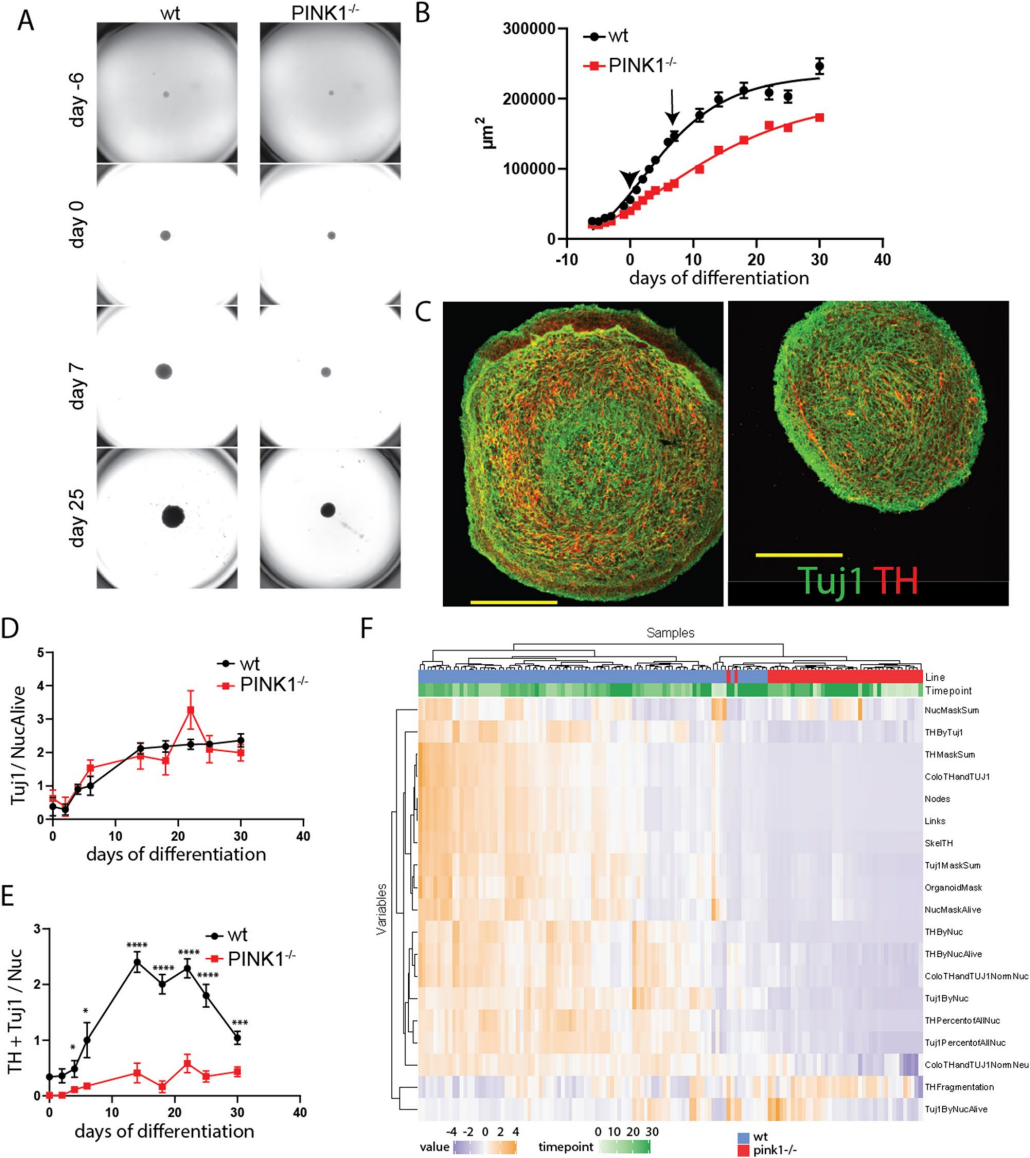
Impaired dopaminergic differentiation in human *pink1*^*-/-*^ midbrain-specific organoids. (**A**) Representative bright field images of midbrain-specific organoids at different time points of differentiation. (**B**) Quantification of the size of the organoids by measuring their area in the images shown in (**A**). Arrow head indicates start of differentiation and arrow indicates start of maturation. Data points represent mean ± SEM. N = 3 independent generations of organoids, each starting with 48 organoids/line. (**C**) Representative images of day 30 midbrain-specific organoids sections (wt left, pink1-/- right) stained for Tuj1 (green) and TH (red). (**D**) Quantification of neuronal content of organoid sections measured as sum of Tuj1 positive pixels (neuronal marker) normalized by Hoechst positive pixels (nucleus marker) at day 0, 2, 4, 6, 14, 18, 22, 25, and 30 of differentiation. (**E**) Quantification of dopaminergic neuronal content measured as sum of Tuj1/TH double positive pixels normalized by Hoechst positive pixels. Data points represent mean ± SEM. P values are calculated by two-tailed Mann–Whitney-test. N = 3 independent generations of organoids, *p < 0.05, ***p < 0.001 ****p < 0.0001. (**F**) Heatmap displaying the features extracted from the image analysis and cluster analysis.

To test whether the impairment of DA neurogenesis caused by PINK1 deficiency can be replicated in a human cellular model, we generated isogenic midbrain-specific organoids from small molecule derived neural progenitor cells (smNPC) as previously described (see methods section). After six days of spheroid formation, organoids were subjected to differentiation medium for six days and subsequently cultured in maturation medium for 24 days. The size of the organoids was monitored over time and revealed a reduced growth rate of *PINK*^*-/-*^ organoids compared to wild type organoids (Fig. 7A). Nonlinear curve fitting using Gompertz growth model confirmed that the growth curve of the two lines differ significantly (*P* < 0,0001) (Fig. 7B). Despite the reduced growth of *pink*^*-/-*^ organoids, immunocytochemical analysis of *Tuj1* expression, when normalized to the nuclear staining with HOECHST, demonstrated that global neuronal differentiation in organoids of both lines was similar (Fig. 7C,D). However, from day four of differentiation, *PINK1*^*-/-*^ organoids showed a significantly reduced proportion of *Tuj1/TH* double positive DA neurons over time compared to the isogenic controls (Fig. 7E), indicating impairment of DA neuronal differentiation in the absence of PINK1. Properties such as morphology and type of the neurons were extracted from the nuclear (HOECHST), neuronal (Tuj1), and DA (TH) staining and several features were calculated based on this automated image analysis (for detailed description see materials and methods). A high level view of all features that were extracted from the image analysis illustrated that organoid sections predominantly cluster in a line-specific (Fig. 7F) rather than a time point-specific manner, revealing a genotypic difference in DA differentiation efficiency.

## Discussion

Post-embryonic neurogenesis has been observed in several niches within the vertebrate brain, including the dentate gyrus and the subventricular zone in mammals, and in each niche, appears to decline with age^40–44^. Zebrafish are an ideal vertebrate model to study neurogenesis due to their external development and larval brain transparency and the comparative ease of assessing adult neurogenesis at multiple time points. Our study shows, for the first time, that two populations of Th1^+^ DA neurons of the zebrafish rostral posterior tuberculum, those of the TPp and the PVO, are generated into adulthood in a manner that declines with age. Importantly, the axons of DA TPp neurons ascend to the subpallium, and are therefore thought to be functionally equivalent to mammalian ascending nigrostriatal DA neurons. Most intriguingly, our data shows that in a robust vertebrate model of Pink1 deficiency, the *pink1*^*-*/-^ zebrafish, Th1^+^ neurons of the TPp and PVO are found in comparable numbers to those in young adult wild-type fish, but thereafter showed a significant decline.

Isolated observations in animal models of PD always raise concerns about the applicability of any results to human patients with this condition. However, the observation of impairment of DA neurogenesis in a PINK1-deficient, human tissue derived organoid model confirmed the initial observations. Future studies need to confirm whether the observed effect of PINK1 deficiency is specific for DA neurogenesis or may also affect other neuronal subpopulations such as serotoninergic neurons. However, the lack of an effect of PINK1 deficiency on global neurogenesis in both our zebrafish studies (Fig. 5C + D) and our human organoid model (Fig. 7C + D) is at least suggestive of a preferential effect of PINK1 deficiency on DA neurons. The observations of a growth deficit of PINK1 deficient smNPC-derived midbrain specific organoids are also in line with the results of a previous study that identified an interaction of NOTCH and PINK1 at the mitochondria via a noncanonical pathway, and showed that PINK1 deficiency in human NSC significantly inhibited their proliferation^17^. Impaired growth was also observed in murine PINK1 deficient NSC and linked to mitochondrial dysfunction^18^. These *Pink1*^*-/-*^ NSC displayed in vitro elevated glycolysis but a reduced maximum respiratory capacity and reduced spare respiration capacity. Spare respiratory capacity is crucial in times of high metabolomics demand like during differentiation. Indeed, initiation of differentiation of adult stem/progenitor and induced pluripotent stem cells requires a metabolic switch from glycolysis to mitochondrial oxidative phosphorylation^45^. The start of differentiation in our organoid model coincided with an increasing divergence of the growth curves for the two isogenic lines. Although the different growth rates suggest a quantitative impairment in neurogenesis in the PINK1 deficient line, it does not reflect a qualitative difference in neuronal differentiation—the relative number of neurons (when normalized to cell number [nuclear staining]) per organoid and time point is comparable between the wt and *PINK1*^*-/-*^ lines. The number of Tuj1 + neurons increased during the first 14 days of differentiation and maturation in both wt and *PINK1*^*-/-*^ organoids. From there on until the end of the experiment at day 30 the overall number of neurons remained stable indicating the lack of neurodegeneration during that period of time in both lines. However, the differentiation into Tuj1 + /TH + neurons was significantly reduced in the PINK1 deficient line suggesting a specific impairment of dopaminergic differentiation in the absence of PINK1 in midbrain-specific organoids.

Together our findings strengthen the idea that factors that impact on de novo neuronal generation may contribute to the pathology of PD, and raise the possibility that, even in non-regenerative mammals, loss of function of the *PINK1* gene may result in a reduced number of DA neurons in early postnatal life stages. A recent detailed study undertaken on human hippocampal neurogenesis identified preserved neurogenesis in healthy human individuals from 14 to 79 years and thus also challenges the previous assumption of absent neurogenesis in aging humans^46^.

What mechanisms might cause reduced neurogenesis in the absence of PINK1? In mouse, a recent study suggests that loss of PINK1 results in deficits in mitochondrial function in neural stem cells (NSC)^18^. The differentiation of NSCs to mature neurons is accompanied by a metabolic switch which shifts NSCs from predominantly glycolytic metabolism into a metabolic state which is highly dependent on mitochondrial oxidative phosphorylation. This progressive shift in metabolism from NSCs to postmitotic neurons is paralleled by changes at the level of metabolic enzyme expression. This metabolic shift does not only alter the source of ATP but is also an important factor in cell cycle regulation and proper neuronal differentiation^18^. Reduced oxidative phosphorylation with marked impairment of the mitochondrial respiratory chain has been observed both in *PINK1* mutant patient tissue and a wide range of PINK1-deficient model systems, including our *pink1*^*-/-*^ zebrafish model^8^ and may be the key mechanism for impaired neurogenesis in PINK1 deficiency. However, other factors also need to be considered. Mitochondrial dynamics, in particular the balance of fusion and fission, are crucial to regulation of neural stem cells: a recent report suggests that mitochondrial fusion is necessary for the maintenance of NSC self-renewal^47^. Changes in mitochondrial shape have the capacity to direct the metabolic changes that occur during neurogenesis and regulate the fate of NSCs^18^. Lack of functional PINK1 may tip the balance of mitochondrial fusion/fission dynamics toward more fusion^48–50^. Thus, loss of functional PINK1 could push NSCs toward self-renewal at the expense of generation of progenitors. Impaired mitochondrial function is a unifying feature of both sporadic and all genetic forms of PD, but probably particularly relevant in the pathogenesis of early onset PD due to *parkin* or *PINK1* mutations^51^. Changes in mitochondrial morphology as well as abnormal function of the mitochondrial respiratory chain—as observed in PINK1 deficiency—can also both result in increased levels of mitochondrial reactive oxygen species (ROS). Increases in mitochondrial ROS levels can commit NSCs to progenitor fate^18^. Future studies need to determine whether the observed effect of PINK1 on adult DA neurogenesis is due to a single impaired mechanism such as impaired mitophagy or whether combined impairment of several mechanisms underpin the impaired adult DA neurogenesis in PINK1 deficiency.

Our observations add weight to recent speculation that an age-related, or genetic predisposition to decline in de novo neurogenesis could contribute to neurodegenerative diseases^52–56^.

## Materials and methods

### Zebrafish husbandry

Adult and larval zebrafish were housed at The Bateson Centre, University of Sheffield; experimental procedures were in accordance with UK Home Office Animals (Scientific Procedures) Act 1986 (Project license number: PPL70/8437, Professor Oliver Bandmann. Personal license: PILI5B38EB05, Sarah Brown). Zebrafish were housed in tanks at a density of no more than four zebrafish per litre, at a constant temperature of 28 °C and on a 14-h light, 10-h dark cycle. The *pink1* mutant zebrafish (*pink1*^*sh397/sh397*^, https://zfin.org/ZDB-FIG-140514-10) was generated with TILLING mutagenesis and has been reported elsewhere (8). For procedures, adult zebrafish were anaesthetized using tricane (PharmaQ Hampshire) (0.016% w/v). For adult brain dissections, zebrafish were anaesthetized with a stronger solution of tricane (0.4% w/v) to allow immediate anesthesia, and culled by decapitation.

We confirm that all experimental protocols were reviewed by the University of Sheffield Project Applications and Amendments Committee (PAAC) and approved by the UK Home Office (Project license number: PPL70/8437, Professor Oliver Bandmann). We also confirm that all experiments were carried out in compliance with the ARRIVE guidelines (https://arriveguidelines.org).

### Adult EdU injections

For EdU analyses, adult zebrafish were anaesthetized with tricane (0.016% w/v) and injected intraperitoneally with 5μl of 10 mM EdU (C10339; ThermoFisher Scientific) in HBSS (14,025–092; ThermoFisher Scientific) once daily for 3 days. After an additional 5 days, zebrafish were sacrificed and brains were collected .

### Conditional lineage tracing

Tg(her4:ERT2CreERT2) and Tg(ubi:loxGFPloxmCherry) transgenic lines^37,57,58^ were incrossed to create the Tg(her4:ERT2CreERT2); Tg(ubi:loxGFPloxmCherry) line. To induce recombination, 5μl of 10 mM tamoxifen (T58) diluted 1:5 in sunflower oil was injected intraperitoneally once and zebrafish were replaced in tanks and chased for 32-days. A control group was injected with oil alone alongside experimental animals. No recombination events were observed in control animals. After the chase period, zebrafish were sacrificed and brains were collected.

### Immunohistochemistry, fluorescent in situ hybridisation and EdU detection

Serial sections throughout the DC1-DC4 populations of adult and embryonic zebrafish brains were labelled using anti-Th1 (22,941; Immunostar) at 1:1000, anti-cleaved caspase-3 (9661; Cell Signalling) at 1:400 or anti-Otp at 1:400^59^ and counter-stained with DAPI. Sections were labelled with secondary antibodies; Alexa 488 or Alexa 594 (Molecular Probes). For Th1 + EdU + analyses, EdU + cells were analysed in serial sections throughout each population using the Click-iT™ EdU Alexa Fluor™ 594 Imaging Kit (C10339; ThermoFisher Scientific). Fluorescent in situ hybridisation was achieved using the tyramide amplification kit (B40915, ThermoFisher Scientific), the riboprobe was *rx*3^60^.

### Image acquisition and statistical analysis

Images of adult brain sections were acquired using an AxioImager.Z1 with Apotome (Zeiss), and Axiovision 4.8 software. Z-stacks were taken and processed to give a maximum intensity projection (MIP) or to generate the 3-d views. 3-d rendered videos were prepared using FIJI 3D viewer. Images were processed in Photoshop (CC; Adobe) and cells were quantified using the Photoshop count tool. Images show single representative sections, but counts were of the entire populations; all quantitative analyses were performed in a blinded manner and individual neurons assigned on the basis of co-localisation to DAPI-stained nuclei. Prism 7 was used to perform statistical analyses and to generate graphs. Statistical significance was testing using unpaired t-tests for comparison between two groups, and Analysis of Variance (ANOVA) with post hoc analysis via Tukey’s test was used to test for differences among more than two groups. In all cases, standard deviation is reported and significance values are denoted as follows; Not significant *p* > 0.05, *p < 0.05, **p < 0.01, ***p < 0.001.

### Generation of organoids

Organoids were generated on the automated platform of the Disease Modelling and Screening Platform (DMSP) at the LCSB. Based on a previously published protocol^61^, a suspension of smNPC with a concentration of 30,000 cells/ml was loaded onto the platform and 100 µl/well were dispensed into Ultra-Low Attachment Multiple 96-well plate (Corning® Costar®). An isogenic PINK1 homozygous knockout line was made using TALEN directed to PINK1 exon 1 causing complete loss of PINK1 mRNA and protein. The TALEN was directed in iPSCs from a healthy female. The reprogramming of the healthy, isogenic control fibroblasts to iPSCs has been previously described and the cell line fully characterised^62^. Columns 1—6 of the plate was loaded with wt smNPC and columns 7 – 12 with *PINK1*^*-/-*^ smNPC and subsequently centrifuged for 5 min at 300 g to collect cells at the bottom centre of the well to facilitate aggregation. The cells were kept for spheroid formation for 6 days in smNPC medium as previously described^62^. Organoid differentiation was induced by subjecting the spheroids for 6 days to differentiation medium (DMEM-F12 (Invitrogen)/Neurobasal (Invitrogen) 50∶50 including N2 supplement 1∶200 (Invitrogen), B27 supplement lacking vitamin A 1∶100 (Invitrogen), 1% penicillin/streptomycin/glutamin, supplemented with 1 µM Purmorphamine, 10 ng/ml BDNF, 10 ng/ml GDNF, 1 ng/ml TGF-β3 (all PeproTech), 500 µM Dibutyryl-cAMP (Santa Cruz)). On day 7 Purmorohamine was withdrawn from the medium and organoids were cultured for another 24 days for maturation. At indicated timepoints, 3–4 organoids per line were collected and fixed over night in 4% PFA at 4° C under shaking condition. After 3 washes with PBS (15 min), organoids were embedded in 3–4% low-melting point agarose in PBS. Embedded organoids were sectioned with a vibratome (Leica VT1000s) into 40 µm sections and stained as previously described (https://doi.org/10.1038/s41531-019-0078-4).

### Image analysis of organoid staining

The image analysis was performed using MatLab, The MatWorks Inc. as previously described^63^. Briefly, a frequency-domain filtering was applied the channels containing neuronal information by doing a Fast Fourier transform, and a high pass Butterworth filtering for sharpening the images. The cut-off frequency was set to 7 and the order of the filter to 1. Afterwards, images were transformed back to the spatial domain, and structures selected by intensity and size. For the channel containing nuclear information, a difference of Gaussians was performed by subtracting a convoluted foreground (with a filter of size 101 pixel, and standard deviation of 3 pixel) with a convoluted background one (with a filter of size 101 pixel, and standard deviation of 7 pixel). Positive structures were further selected by intensity and size. Properties related to the morphology of the neurons were extracted via skeletonization. Due to the compact structure of the organoids, an approach for estimating the percentage of cells belonging to the different types of neurons was performed by further splitting the nuclei mask with the watershed function. Evaluation of the type of neuron was determined by the pixel signal at the perinuclear area of these newly segmented nuclei. Once the different masks were generated, several features were extracted from them. Those derived from the sum of pixels of the masks were NucMaskSum, Tuj1MaskSum, THMaskSum, OrganoidMask. Looking at the size and intensity of the nuclear structure, pixels were divided in those belonging to alive or dead cells (pyknotic nuclei presents a reduced size and compacted chromatin) generating the feature NucMaskAlive. Pixel counts were normalized by different features (Tuj1ByNucAlive, THByNucAlive, THByNuc, Tuj1ByNuc, THByTuj1), and colocalization of signal were also calculated and normalized (ColoTHandTUJ1, ColoTHandTUJ1NormNuc, ColoTHandTUJ1NormNeu). Morphometric of the neurons were extracted from their skeleton and classified in SkelTH, Links, Nodes, and THFragmentation as previously described^64^. The different percentage of neuronal types was summarized in the features THPercentofAllNuc and Tuj1PercentofAllNuc.

## Supplementary Information

Supplementary Information 1.

Supplementary Video 1.

Supplementary Video 2.

Supplementary Video 3.

Supplementary Video 4.

Supplementary Video 5.

Supplementary Video 6.

Supplementary Video 7.

Supplementary Video 8.

Supplementary Video 9.

Supplementary Video 10.

Supplementary Video 11.

Supplementary Video 12.

Supplementary Video 13.

Supplementary Video 14.

Supplementary Video 15.
