# Supplementary material for: PINK1 deficiency impairs adult neurogenesis of dopaminergic neurons
Source: Sci Rep. 2021 Mar 23;11:6617. doi: 10.1038/s41598-021-84278-7 (PMC7988014; doi:10.1038/s41598-021-84278-7)
Supplement: Supplementary file 1 — Supplementary Information 1. [file 41598_2021_84278_MOESM1_ESM.pdf]

Supplementary material:

PINK1 deficiency impairs adult neurogenesis of dopaminergic neurons

Sarah J. Brown[1,2,3, #];Ibrahim Boussaad[5,6 #]; Javier Jarazo[7,8 #]; Julia C. Fitzgerald[9], Paul Antony[5]; Marcus Keatinge[1,3,4]; Janna Blechman[10], Jens C. Schwamborn[7,8]; Rejko Krüger[5,11,12]; Marysia Placzek[1,2#]; Oliver Bandmann[1,3#*]

[1]Bateson Centre, University of Sheffield; [2]Department of Biomedical Science, University of Sheffield; [3]Sheffield Institute for Translational Neuroscience (SITraN), Department of Neuroscience, University of Sheffield; [4]Centre for Discovery Brain Science, University of Edinburgh; [5]Translational Neuroscience, Luxembourg Centre for Systems Biomedicine, University of Luxembourg, Luxembourg; [6]Disease Modelling and Screening Platform (DMSP), Luxembourg Centre of Systems Biomedicine, University of Luxembourg & Luxembourg Institute of Health, Luxembourg; [7]Developmental Biology, Luxembourg Centre for Systems Biomedicine, University of Luxembourg, Luxembourg; [8]OrganoTherapeutics SARL, Luxembourg; [9]Hertie-Institute for Clinical Brain Research, University of Tübingen, Tübingen, Germany; [10]Weizmann Institute of Science, Rehovot, Israel; [11]Parkinson Research Clinic, Centre Hospitalier de Luxembourg (CHL), Luxembourg; [12]Transversal Translational Medicine, Luxembourg Institute of Health (LIH), Luxembourg; #These authors contributed equally to the manuscript.

Supplementary figures:

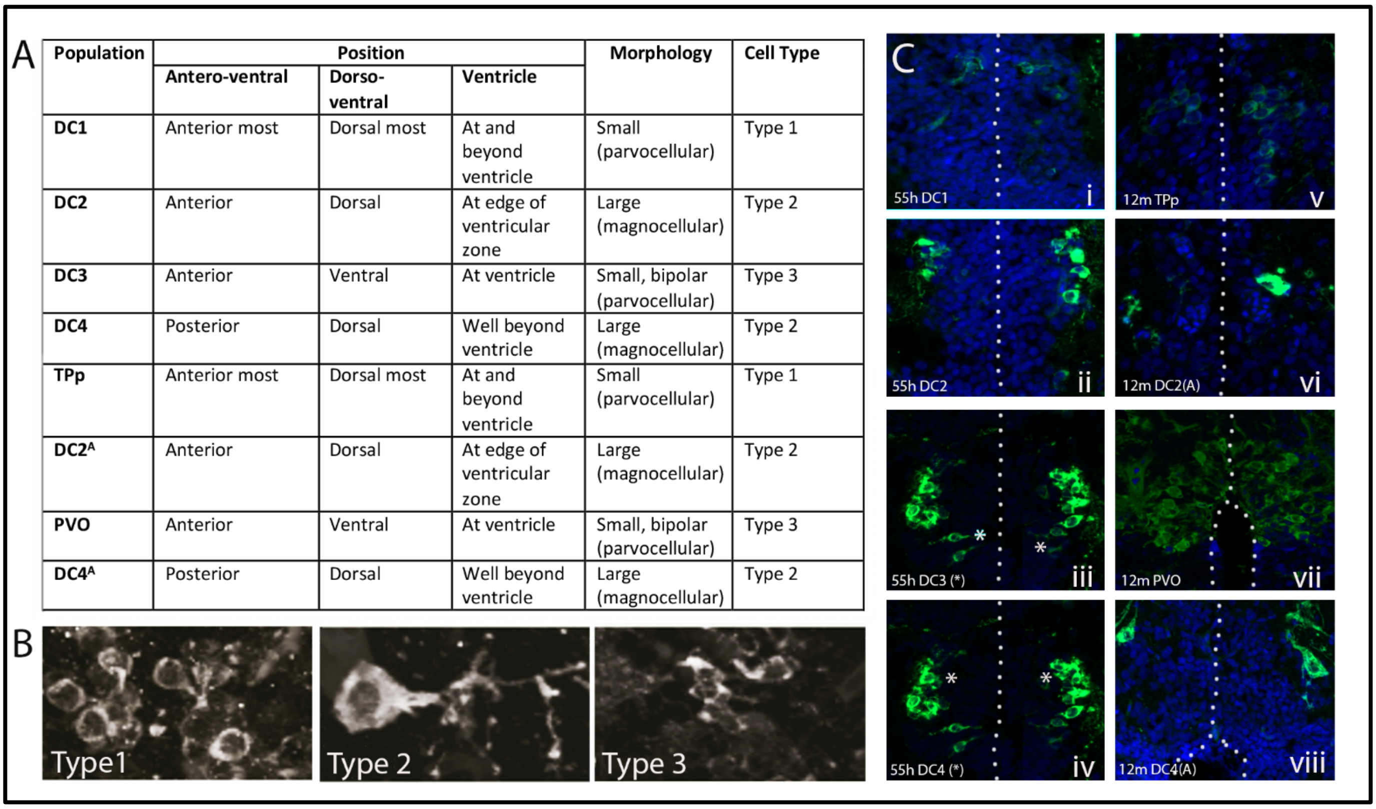

Population	Position			Morphology	Cell Type
	Antero-ventral	Dorso-ventral	Ventricle		
DC1	Anterior most	Dorsal most	At and beyond ventricle	Small (parvocellular)	Type 1
DC2	Anterior	Dorsal	At edge of ventricular zone	Large (magnocellular)	Type 2
DC3	Anterior	Ventral	At ventricle	Small, bipolar (parvocellular)	Type 3
DC4	Posterior	Dorsal	Well beyond ventricle	Large (magnocellular)	Type 2
TPp	Anterior most	Dorsal most	At and beyond ventricle	Small (parvocellular)	Type 1
$DC2^A$	Anterior	Dorsal	At edge of ventricular zone	Large (magnocellular)	Type 2
PVO	Anterior	Ventral	At ventricle	Small, bipolar (parvocellular)	Type 3
$DC4^A$	Posterior	Dorsal	Well beyond ventricle	Large (magnocellular)	Type 2

S1. Characterisation of DA neurons in the embryonic and adult zebrafish PT

(A) Table shows characteristic features of each population, in terms of size, morphology and position relative to dorso-ventral and medio-lateral axes. (B) High power views showing representative morphologies of Type1, Type2 and Type 3 DA neurons. Type 1 neurons are small and rounded; Type 2 neurons are large and have extensive neurites, Type 3 neurons are small and bipolar/elongated. (C) Representative transverse sections of 55hpf and 12m zebrafish, after immunolabelling to detect Th1, to show similarities in morphology and position between embryonic and adult populations. Asterisks point to DC3 neurons (Ciii) and DC4 neurons (Civ). Dotted line indicates ventricle.

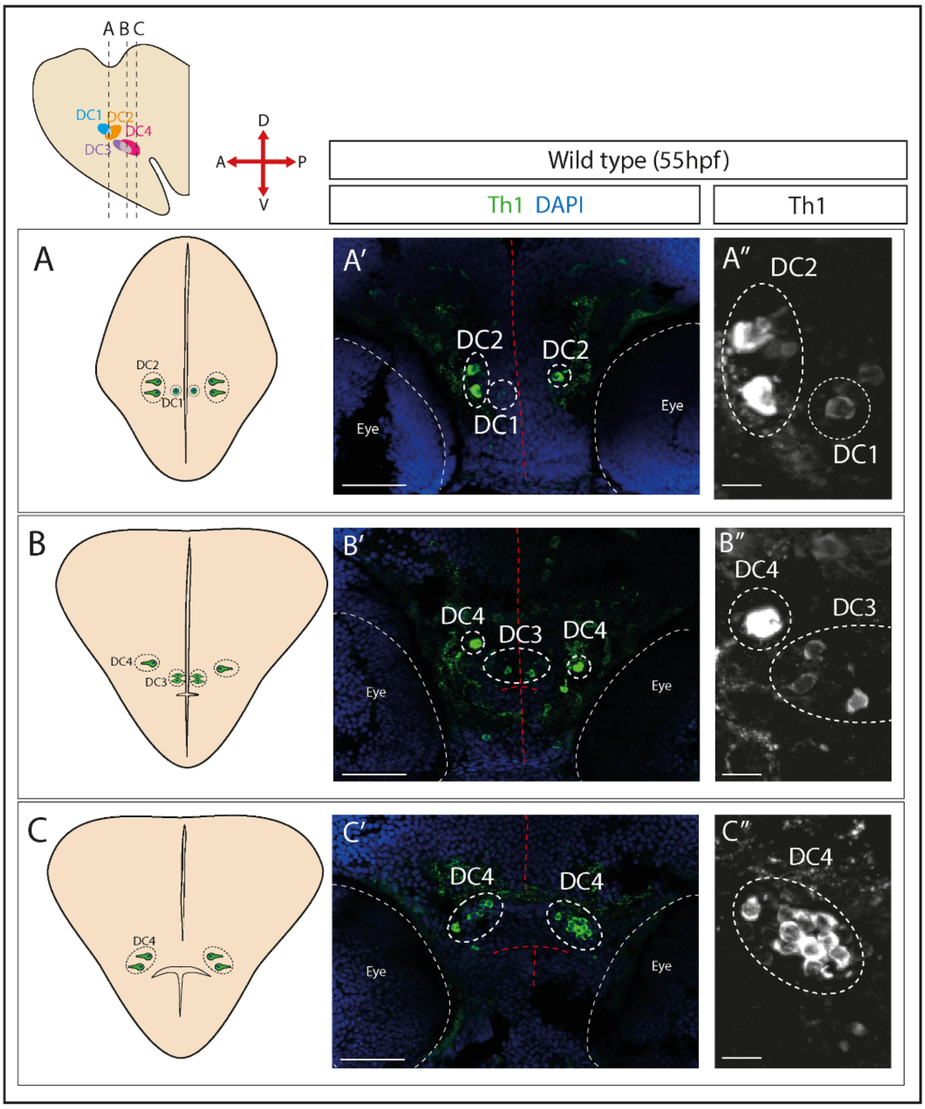

S2: Dopaminergic populations in the embryonic zebrafish rostral PT

(A, B, C). Illustrations showing the orientation and rostral PT population location for transverse planes in A', B', C' and A", B" and C". DA neurosn are represented in green.

(A', B', C'). Fluorescent MIP of a transverse section through a 55hpf PT, labelled with the nuclear marker, DAPI (blue) and Th1 (green). Different populations in the PT are outlined and labelled. Th1 labels DA neurons in distinct populations within the PT. Scale bars: 50µm. Red dotted line indicates ventricle.

(A", B", C"). Fluorescent MIPs for Th1 are shown as magnifications of A', B', C'. Distinct populations in the PT are outlined and labelled. Scale bars represent 10µm.

Small round cells occupy the TPp, large pear shaped neurons form the DC2 and DC4 populations and small bipolar cells occupy the PVO. Scale bars: 10μm.

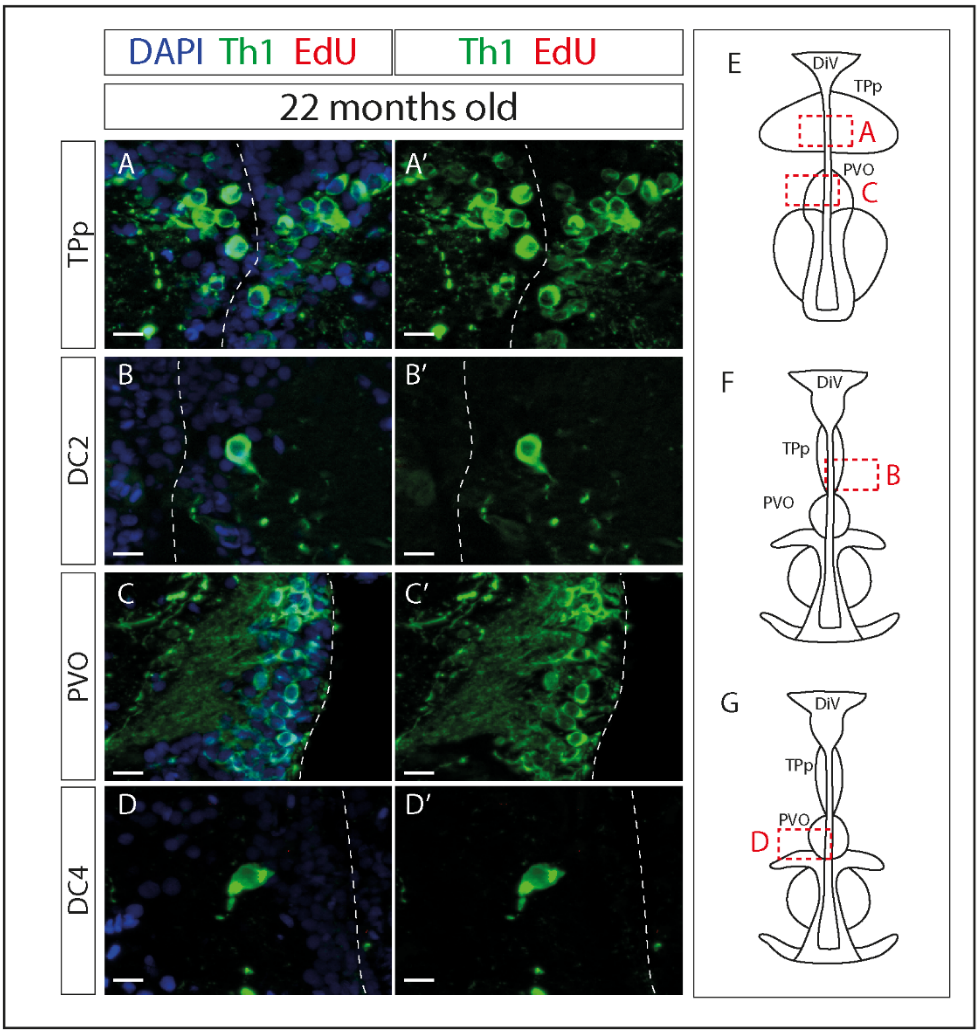

S3. No EdU labelled DA neurons are detected in the rostral PT at 22-months of age. (**A-D'**) Immunohistochemical analysis for Th1 (green), ClickIT™ labeling for EdU (red) and counterstained with DAPI (blue) (A,B,C,D) or shown without DAPI (A',B',C',D',) in transverse sections of 22-month wild type zebrafish brains (n=3). No Th1$^+$EdU$^+$ cells are detected in the TPp (A,A'), the PVO (C,C') or in DC2 (B,B') or DC4 (D,D') populations. Dotted line indicates ventricle. Scale bars: 10µm.

(**E-G**) Schematics indicate the position of images (A-D') within the PT, red boxes indicate position of the corresponding image in relation to the ventricle. Schematics in (E-G) are based on anatomical drawings by Rink and Wullimann [25].

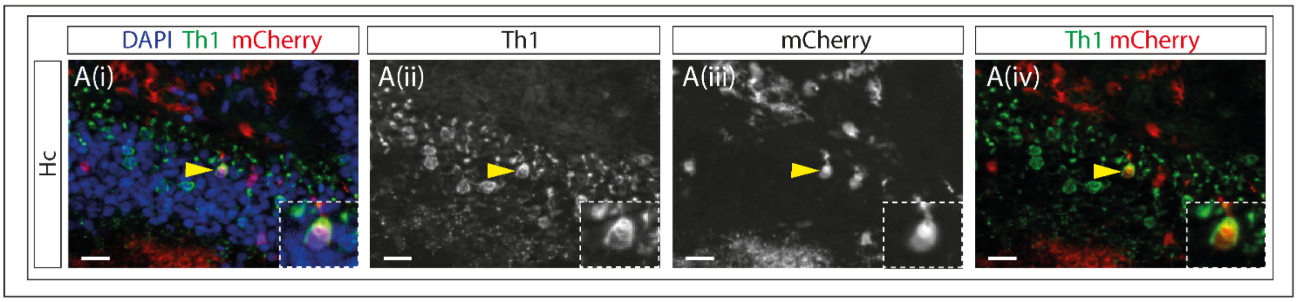

S4. DA neurons in the adult caudal hypothalamus are also generated from Her4-expressing progenitors. (**Ai-iv**) Fluorescent MIP of the caudal hypothalamus of a double transgenic 3-month old zebrafish injected with tamoxifen, labelled with DAPI (blue), Th1 (green) and mCherry (red). Th1 is shown as a single channel in (ii), mCherry is shown in (iii). Th1 (green) and mCherry (red) are shown together in (iv). mCherry+Th1+ cells are observed in the caudal hypothalamus (yellow arrows). Boxed regions show magnified image of the double labelled cell. Scale bars: 10µm.

Supplementary Videos

V1. 3-d rendered image of Z-stacks from the sagittal section shown in Fig 1B': 55hpf embryo, immunolabelled to detect Th1 (green) and counterstained with DAPI (blue).

V2. 3-d rendered image of Z-stacks from the sagittal section shown in Fig 1C': 12m adult, immunolabelled to detect Th1 (green)

V3. 3-d rendered image of Z-stacks showing a zoomed-in view from the transverse section shown in Fig S1(Ci): 55hpf embryo, immunolabelled to detect Th1+ DC1 neurons (green); counterstained with DAPI (blue).

V4. 3-d rendered image of Z-stacks showing a zoomed-in view from the transverse section shown in Fig S1(Cii): 55hpf embryo, immunolabelled to detect Th1+ DC2 neurons (green); counterstained with DAPI (blue).

V5. 3-d rendered image of Z-stacks showing a zoomed-in view from the transverse sections shown in Fig S1 (Ciii and Civ): 55hpf embryo, immunolabelled to detect Th1+ DC3 and DC4 neurons (green); counterstained with DAPI (blue).

V6. 3-d rendered image of Z-stacks showing a zoomed-in view from the transverse sections shown in Fig S1(Cv and Cvi): 12m zebrafish, immunolabelled to detect Th1+ TPp and DC2(A) neurons (green); counterstained with DAPI (blue).

V7. 3-d rendered image of Z-stacks showing a zoomed-in view from the transverse sections shown in Fig S1(Cvii and Cviii): 12m zebrafish, immunolabelled to detect Th1+ DC4(A) and PVO neurons (green); counterstained with DAPI (blue).

V8. 3-d rendered image of Z-stacks from the transverse section shown in Fig 3A (a 3m zebrafish, pulsed with EdU). EdU (red) and Th1 (green) are co-localilsed in TPp neurons (counterstained with DAPI (blue)).

V9. 3-d rendered image of Z-stacks from the transverse section shown in Fig 3C (a 3m zebrafish, pulsed with EdU). EdU (red) and Th1 (green) are co-localised in PVO neurons (counterstained with DAPI (blue)).

V10. 3-d rendered image of Z-stacks from the transverse section shown in Fig 4A(i) (a 3m double transgenic (Tg(her4:ERT2CreERT2); Tg(ubi:loxGFPloxmCherry)) zebrafish, after tamoxifen-recombination). mCherry (red) is detected close to Th1 (green) TPp neurons in the 3m fish, but is not clearly co-localised (counterstained with DAPI (blue)).

V11. 3-d rendered image of Z-stacks from the transverse section shown in Fig 4A(iv) (a 3m double transgenic (Tg(her4:ERT2CreERT2); Tg(ubi:loxGFPloxmCherry)) zebrafish, after tamoxifen-recombination). mCherry (red) is detected close to Th1 (green) TPp neurons in the 3m fish, but is not clearly co-localised.

V12. 3-d rendered image of Z-stacks from the transverse section shown in Fig 4B(i) (a 3m double transgenic (Tg(her4:ERT2CreERT2); Tg(ubi:loxGFPloxmCherry)) zebrafish, after tamoxifen-recombination). mCherry (red) and Th1 (green) co-localise in PVO neurons in the 3m fish; (counterstained with DAPI (blue)).

V13. 3-d rendered image of Z-stacks from the transverse section shown in Fig 4B(iv) (a 3m double transgenic (Tg(her4:ERT2CreERT2); Tg(ubi:loxGFPloxmCherry)) zebrafish, after tamoxifen-recombination). mCherry (red) and Th1 (green) co-localise in PVO neurons in the 3m fish.

V14. 3-d rendered image of Z-stacks from a transverse section taken through the caudal hypothalamus of a 3m double transgenic (Tg(her4:ERT2CreERT2); Tg(ubi:loxGFPloxmCherry)) zebrafish, after tamoxifen-recombination. mCherry (red) and Th1 (green) co-localise in caudal hypothalamic neurons.
